# Numerical study on the characteristics of viscous fingering during the displacement process of non-Newtonian fluid

**DOI:** 10.1371/journal.pone.0309176

**Published:** 2024-09-26

**Authors:** Yu-Ting Wu, Zhen Qin, Huaiyu Ma, Sung-Ki Lyu

**Affiliations:** 1 School of Transportation and Vehicle Engineering, Shandong University of Technology, Zibo, China; 2 School of Mechanical and Aerospace Engineering, Gyeongsang National University, Jinju-si, Republic of Korea; 3 School of Mechanical Engineering, Shandong University of Technology, Zibo, China; 4 Shandong Dayang Mining Equipment Co. LTD, Jining, China; Izmir Katip Celebi University: Izmir Katip Celebi Universitesi, TÜRKIYE

## Abstract

This study uses numerical methods (ANSYS-Fluent) to investigate the viscous fingering of the displaced phase as a shear-thinning fluid in the classic three-dimensional Hele-Shaw cell. Comparing the behavior of fingerings with different properties on the upper and lower surfaces of a three-dimensional model, it was found that when the upper and lower surfaces are walls, under the combined action of moving contact lines and Saffman-Taylor instability, fingering splitting occurs at the tip, resulting in the appearance of two fingers at the interface. In addition, we have found that interfacial tension has a suppressive effect on short waves. As the interfacial tension increases, the velocity at the advancing tip decreases. Therefore, when the interface tension is 0, viscous fingering displacement reaches the farthest distance. We have also conducted research on the viscous fingering at different temperatures. The results indicate that increasing the temperature leads to a decrease in the viscosity of the displaced phase, making the flow more stable. As the temperature rises, the pressure gradient inside the flow path increases, pushing the viscous fingering further.

## 1. Introduction

Viscous fingering refers to the unstable phenomenon that occurs at the displacement front when a low-viscosity fluid displaces a non-miscible high-viscosity fluid. Due to the non-uniform advancement of the two-phase interface resembling a ‘finger-like’ morphology, this process is referred to as fingering [1–3]. Viscous fingering is a common phenomenon in various fields of multiphase flow, such as nature, daily life, and industrial production, including oil extraction [4], geothermal reservoir recharge [5], drug injection [6] and so on. This instability is an undesirable phenomenon as it reduces the sweep efficiency and lead to a decrease in displacement efficiency [7–9]. On the other hand, the instability of the interface benefits chromatographic separation, improving mixing in non-turbulent systems and small-scale devices [10]. Choosing between a stable or unstable interface based on the application is crucial for ensuring the stability or instability of the control interface.

Saffman and Taylor [11] first analyzed the influence of interfacial tension on the viscous fingering in the flow field, leading to extensive experimental and theoretical research by many scholars on this issue [12–16]. One commonly used device for observing the advancement of a viscous fingering is the Hele-Shaw cell (HSC). Dong et al. [17] studied a series of factors related to finger formation in a two-dimensional channel using numerical methods. Especially, they pay attention to the impact of gravity and have found that under the same wetting conditions, the presence of gravity shortens the penetration time. Kang et al. [18] simulated the displacement of a viscous fluid by another fluid in a two-dimensional channel, discovering that the capillary number and wetting properties affect the width and length of fingerings. Shi et al. [19] conducted a numerical study on the phenomenon of viscous fingering in channels using lattice Boltzmann method. Studied the effects of capillary number, viscosity ratio, wetting properties, gravity acceleration, three-dimensional (3D) geometry, and non-Newtonian rheological characteristics on finger formation. The finger pattern generated by three-dimensional geometric shapes differs significantly from the patterns generated by two-dimensional (2D) geometric shapes, and the width of the finger being influenced by the three-dimensional geometric shapes and channel wall wettability. However, few studies have focused on the impact of the viscous fingering on the upper and lower wall surfaces in three-dimensional models when the displaced phase is a non-Newtonian fluid.

Grosfils et al. [20] studied the instability of interfaces, the influence of surface tension, and the reactivity between two fluids. Bischofberger et al. [21] studied the viscous fingering and its stability of two miscible fluids. In this study, the surface tension was set to zero because it can prevent short-wave fluctuations. They discovered that instability is measured by unstable wavelength, which depends on viscosity ratio. Azaiez et al. [22] investigated the linear interface stability of non-Newtonian fluids using numerical analysis with the finite difference method. They observed that the shear-thinning effect is sufficient to cause significant changes in flow instability. Their conclusion is that shearing thinning (non-Newtonian) fluids are less stable than Newtonian fluids. However, few have explored the impact of the change in interfacial tension on the viscous fingering when displaced by a non-Newtonian fluid.

Compared to Newtonian fluids, non-Newtonian fluids receive less attention in the flow through HSC. However, it is worth noting that fluid rheology plays a crucial role in the evolution of viscous fingering. The viscous fingering is the flow instability caused by the viscosity difference of two liquids, where even a very small disturbance can lead to significant movement of the contact line. Logvinov et al. [23] found that the diffusion rate of the fluid strongly depends on the power law index n. Qin et al. [24] utilized numerical simulation methods to study non-Newtonian fluids under air displacement with different power-law indices. The research revealed that for lower power-law indices, the elongated fingers are shorter and thicker, leading to higher displacement efficiency. Additionally, numerous scholars have conducted extensive research on the viscous fingering in radial Hele-Shaw cells, revealing that shear-thinning fluids are more prone to instability than both Newtonian and shear-thickening fluids [25–27].

In summary, in this study, we will analyze the viscous fingering in the process of air displacing shear-thinning fluids in a three-dimensional HSC model using numerical simulation methods. The expression of parameters used is shown in Table 1. Investigating the influence of wall surfaces on viscous fingering in three-dimensional models, comparative analysis was also conducted for different surface tensions. In addition, the development of the viscous finger under different wall temperatures has also been studied.

**Table 1:** Parameters.

Parameter	Symbol
Hele-Shaw cell	HSC
Three-dimensional	*3D*
Two-dimensional	*2D*
Fluid volume method	VOF
Continuum surface force	CSF
Pressure	*p*
Velocity vector	***u***
Density	*ρ*
Dynamic viscosity	*μ*
time	*t*
unit vector	***I***
Surface tension	***F***_*st*_
Gas phase	*a = g*
Liquid phase	*a = l*
Volume fraction	*r*
Gravity	$\vec{g}$
Shear rate	*γ*
Power law exponent	*n*
Number of capillaries	*C*_*a*_
Velocity at the fingering tip	*ν*
Temperature	*T*
Temperature dependence	*H(T)*

## 2. Numerical method

In the HSC system, the governing equations are typically expressed using the Darcy’s law for depth-averaged flow, where the velocity of each fluid component is averaged through the depth, as given by Eq (1).


$$u_{j}=-\frac{h^{2}}{12\mu_{j}}\nabla p_{j}$$ (1)

Where *p*_*j*_ and ***u***_*j*_ are the phase *j* depth-averaged pressure and velocity vectors, respectively.

Furthermore, the fluid velocity is generally small, and the flow can be considered incompressible, so the continuity equation can be changed to Eq (2)

$$\nabla ∙u_{j}=0$$ (2)

In the equation, ***u*** represents the velocity vector, and its equation of motion is

$$\rho \left[\frac{\partial u}{\partial t}+(u∙\nabla )u\right]=\nabla ∙(-pI+\mu [\nabla u+{\left(\nabla u\right)}^{T}])+F_{st}$$ (3)

In the formula, *p* represents pressure, *ρ* represents density, *μ* represents dynamic viscosity, ***I*** represents unit vector, *t* represents time, ***F***_*st*_ represents surface tension.

In this study, we utilized ANSYS Fluent for numerical simulation. In order to capture the two-phase interface and depict the topography of viscous fingering, the volume of fluid (VOF) method based on a fixed Euler computational mesh was used to trace the free boundary. The method assumes that the fluids involved in the modeled flux are immiscible, but are included in the computational mesh, and the VOF method also uses a single coupled pressure equation and a single system of momentum equations for each dimension. In a modeled multiphase flow, it generates a shared velocity field, and the mass-weighted mean mass and momentum transport equations are the basis for modeling. These equations where there is no phase-transfer of mass are defined as

$$\frac{\partial }{\partial_{t}}(r_{\alpha }\rho_{\alpha })+\nabla .(r_{\alpha }\rho_{\alpha }\vec{U_{\alpha }})=0$$ (4)

$$\frac{\partial }{\partial_{t}}(r_{\alpha }\rho_{\alpha }\vec{U_{\alpha }})+\nabla .(r_{\alpha }\rho_{\alpha }\vec{U_{\alpha }}⊗\vec{U_{\alpha }})=\nabla .\left(r_{\alpha }\mu_{\alpha }(\nabla \vec{U_{\alpha }}+{\left(\nabla \vec{U_{\alpha }}\right)}^{T})\right)-r_{\alpha }\nabla p+r_{\alpha }\rho_{\alpha }\vec{g}+\vec{F_{st}}$$ (5)

Where the subscript *α* refers to the gas (*g*) and liquid (*l*) phases such that *r*_1_+*r*_*g*_ = 1. In addition, $\vec{U_{\alpha }}$ represents the velocity field of phase *α*,*r*_*α*_-volume fraction, *ρ*_*α*_-density, *p*-pressure, *μ*_*α*_-viscosity, $\vec{g}$ -gravity and $\vec{F_{st}}$ represents the surface tension.

**Fig 1:**
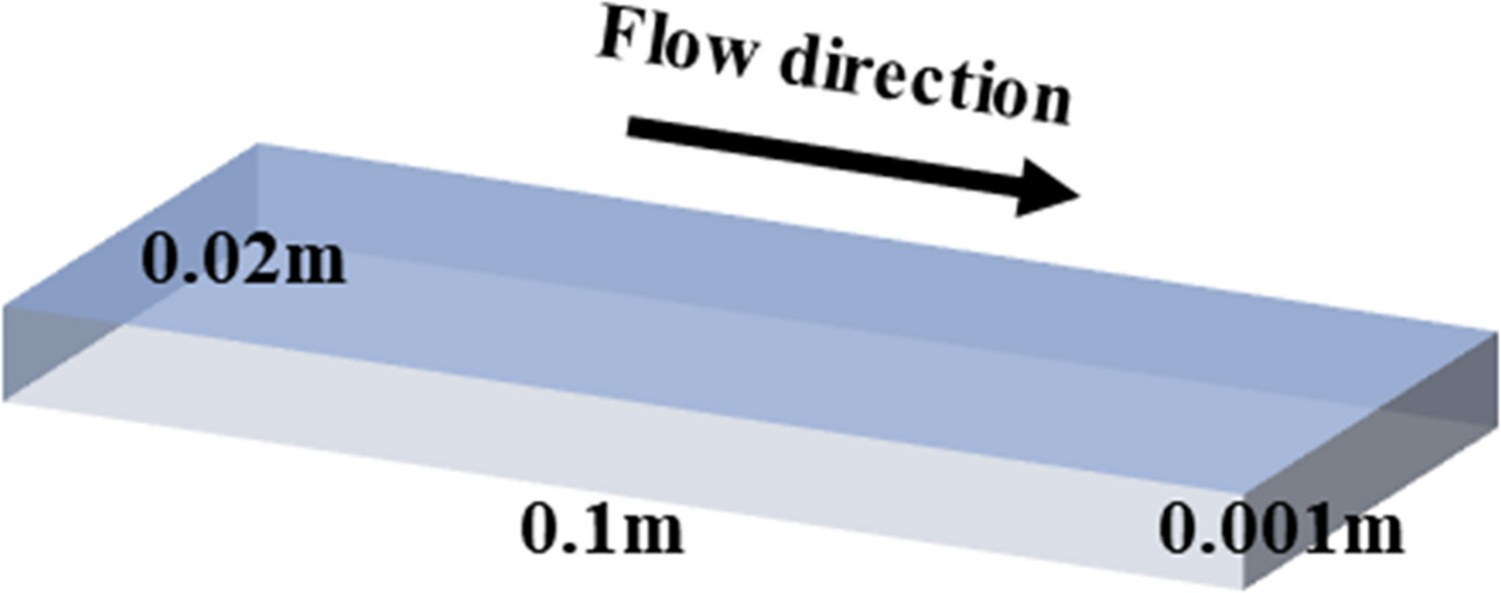
3D model of HSC.

**Fig 2:**
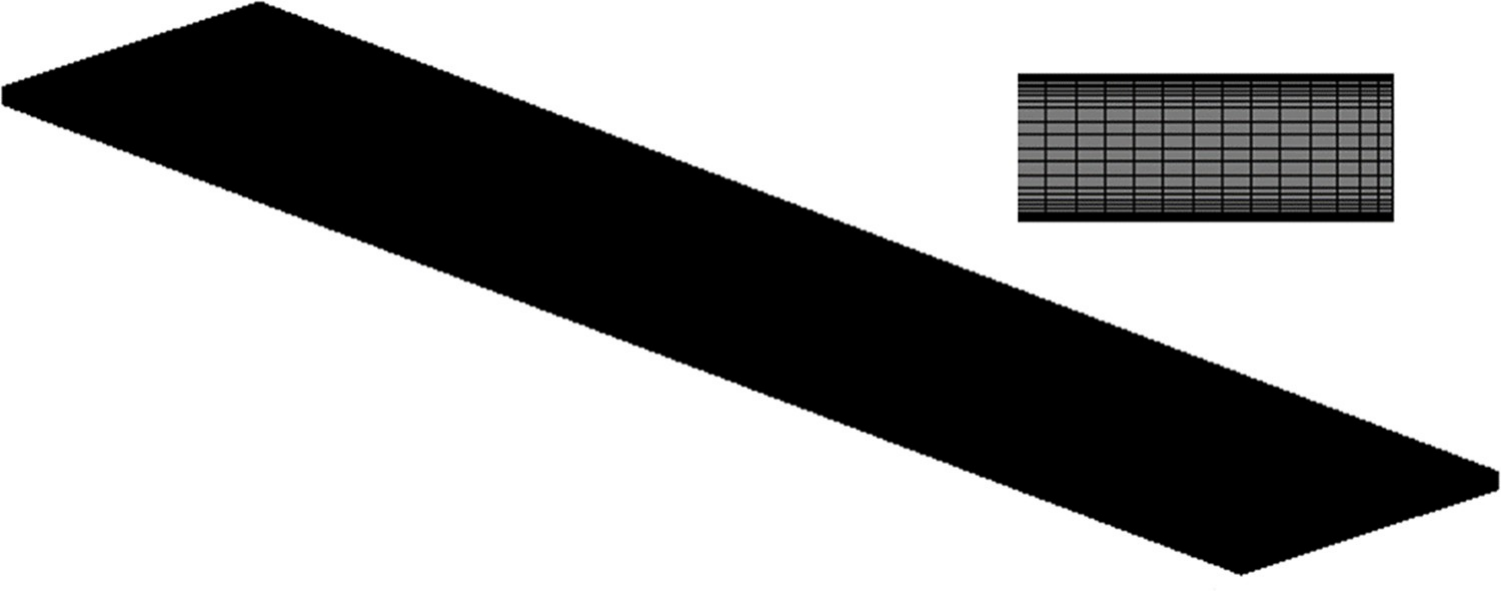
Grid of the computational domain.

In this study, based on 3D rectangular HSCs, which are 10 cm in length and 2 cm in width, respectively, and the distance between the upper and lower plates is 1 mm, as shown in Fig 1. Throughout the simulation, a high-quality structural mesh was used, as shown in Fig 2, with a mesh count of about 1.4 million, and the mesh was encrypted at the boundary layer to improve the accuracy of the simulation. In the grid independence verification, the outlet velocity was used as the criterion. It was found that when the grid count exceeds 1.4 million, the outlet velocity ceases to vary with further increases in grid count. Therefore, the grid count was approximately determined to be 1.4 million [24].

In the setting of the boundary conditions, the air displaces the non-Newtonian fluid at a constant velocity of 0.02 *m/s*, and the outlet is the average static pressure outlet condition of the ambient atmosphere, which is *P*_*rel*_ = 0 Pa. For non-Newtonian fluids in the displaced phase, the viscous fingering of shear-thinning fluids with n = 0.4 is mainly considered. In numerical simulations, a non-Newton power-law model of shear-thinning fluids is used.

$$\mu =\mu_{0}\gamma^{n-1}$$ (6)

where *γ* is the shear rate, and *n* is the power law exponent. The fluid is classified into shear thickening for *n*>1, shear thinning for *n*<1, and the fluid recovers the Newtonian behavior at *n* = 1.

## 3. The effect of the plate on the viscous fingering

**Fig 3:**
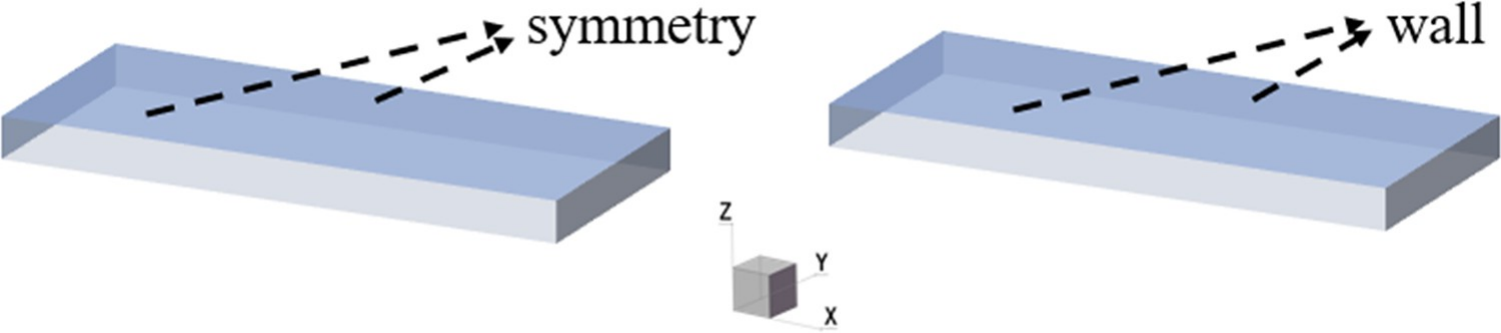
Properties of the upper and lower surfaces.

**Fig 4:**
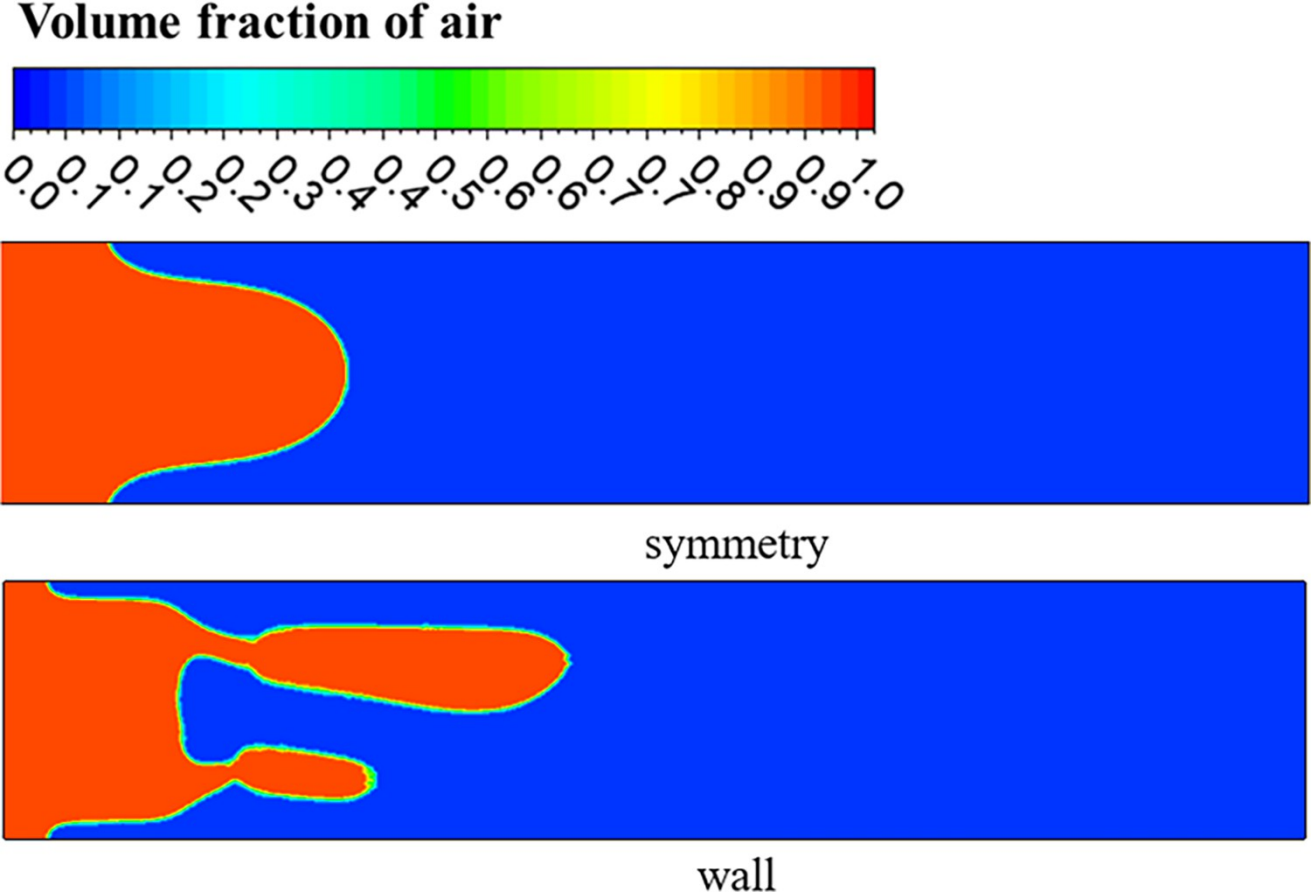
Contour of viscous fingering in *XY* plane.

**Fig 5:**
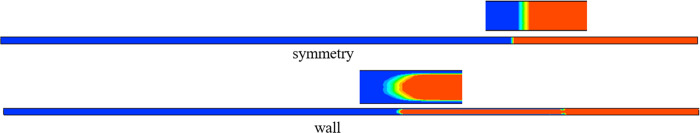
Contour of viscous fingering in plane of *XZ*.

**Fig 6:**
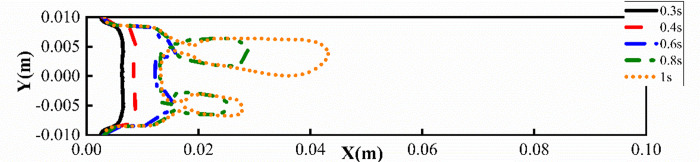
The viscous fingering at different times under the wall on the upper and lower surfaces.

**Fig 7:**
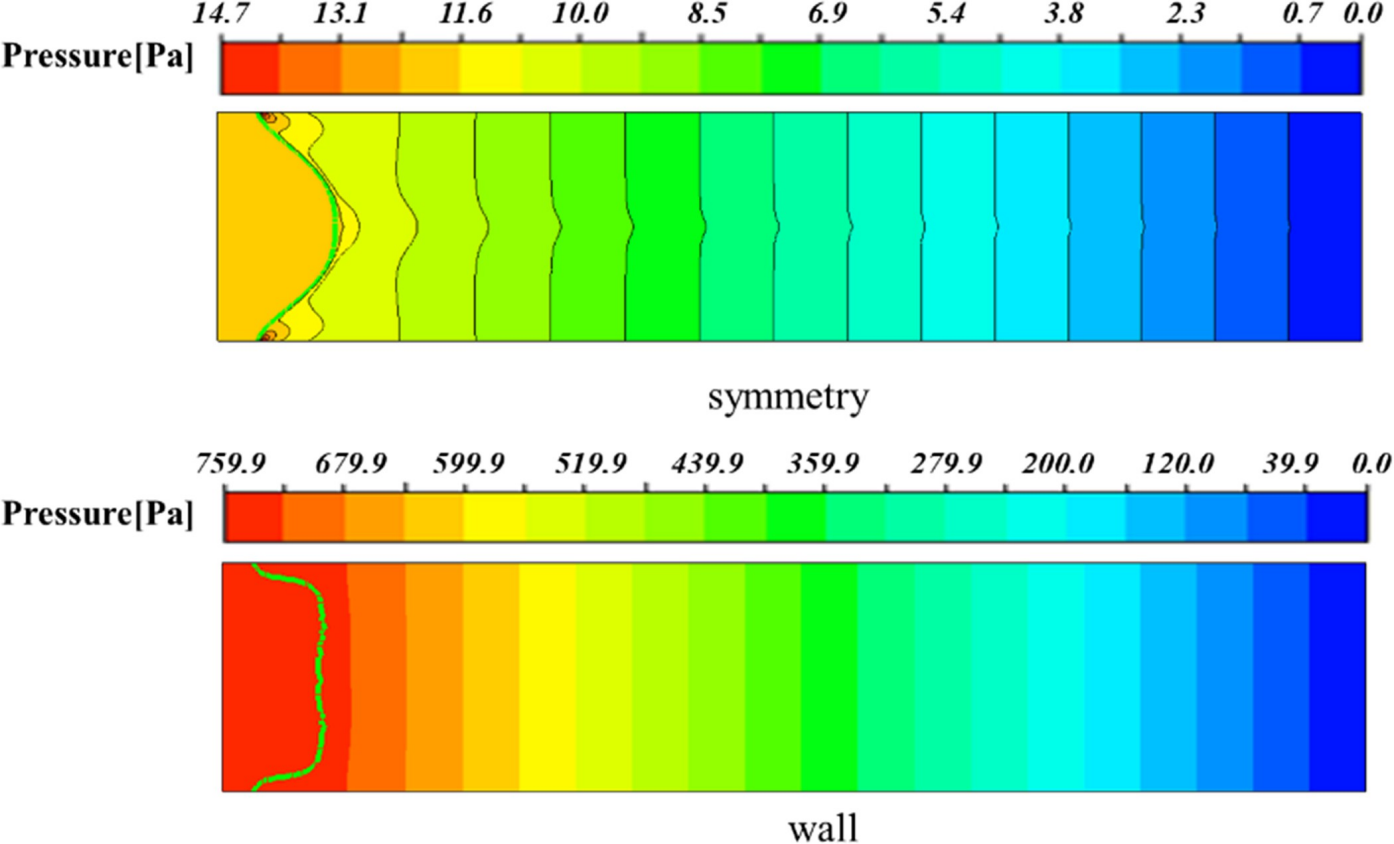
Pressure contour at time 0.4*s*.

In order to study the effect of the plates on the viscous fingering, the upper and lower plates were set to wall and symmetry respectively for comparison, as shown in Fig 3. As can be seen from Fig 4, if the upper and lower surfaces are set to symmetry, the model at this time is similar to a two-dimensional model [28], forming a single and symmetrical finger. When the upper and lower surfaces are set to wall, it can be found that there is a branch-like bifurcation flow, and the interface between the two fluids is an irregular parting feature. It can also be seen from Fig 4 that under the influence of the wall, when the displaced fluid is a shear-thinning fluid, the finger advances split at the tip, and two fingers appear at the interface, and one finger advances faster than the other, which is consistent with the results of Hu [29]. The difference between the presence and absence of walls lies in the need to distinguish between the two fingering processes that occur in the *xy* plane and the *xz* plane. Fig 5 shows a contour plot of two-phase distribution in the *xz* plane. From the figure, it can be observed that during the flow of shear-thinning fluid, the viscosity causes the fluid layer near the wall to experience frictional drag, resulting in reduced velocity. In contrast, regions further away from the wall flow more freely. This phenomenon leads to a ‘bullet-shaped’ flow profile, with faster flow in the center and slower flow near the edges. However, when the upper and lower surfaces are symmetrical, the absence of wall friction allows the front end of the shear-thinning fluid to become flat. In the *xy* plane of Fig 4, the fingering due to the Saffman-Taylor instability occurs when a lower viscosity fluid displaces a more viscous fluid. Therefore, when there is a wall, the morphology between the two phases is the result of a combination of two factors: the moving contact line and the Saffman-Taylor instability. Fig 6 shows the viscous fingering state at different times with the upper and lower surfaces of the wall, and it can be seen that the interface was relatively flat before 0.4s, because the tip of the interface flattened due to the large pressure accumulation in front of the finger. As can be seen from Fig 7, the pressure at the tip of the finger is about 12 Pa when the upper and lower surfaces are symmetry, and about 750 Pa when the upper and lower surfaces are wall. With further displacement, at moment 0.6s, the interface splits to form two almost identical fingers, and subsequently, the two fingers move forward at different speeds and widths. The upper finger moves at about 0.15 m/s, while the velocity of lower finger is decreasing, by 1s, the speed decreased to 0.003m/s. This is due to the fact that the displaced phase is a non-Newtonian fluid, and the shear thinning of the displaced phase makes the finger more unstable, and the finger is more prone to tip splitting, pinching and fusion, which makes the interface more irregular.

## 4. Effect of surface tension on viscous fingering

**Fig 8:**
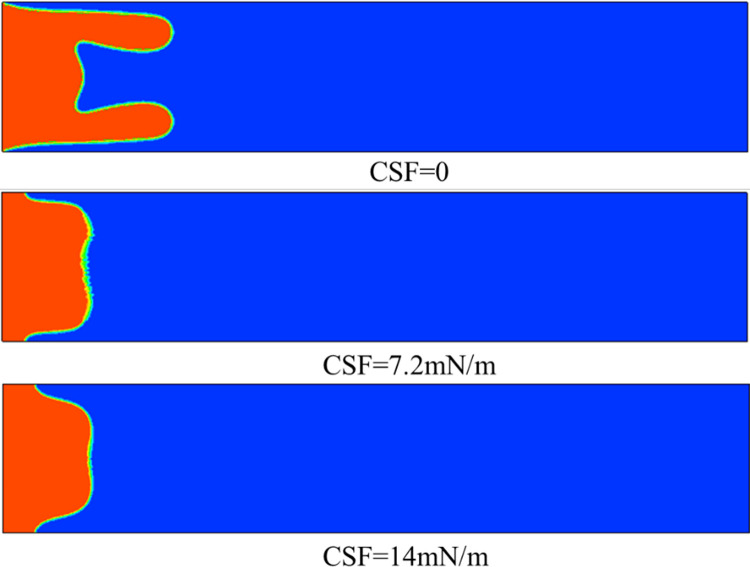
Viscous fingering at 0.5 s under different interfacial tensions.

**Fig 9:**
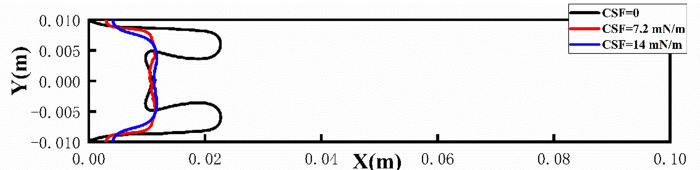
Position of viscous fingering at 0.5s.

The properties of different shear-thinning fluids vary, so it is essential to consider the changes in interfacial tension between these fluids and air. For different surface tensions, the effects of them on viscous fingering were analyzed. The surface tension between the two-phase interface was selected in the continuum surface force (CSF) model, and its values were set to 0, 7.2*mN/m* and 14*mN/m*, where the unit of surface tension is millinewton per meter (*mN/m*). As can be seen from Fig 8, when the interfacial tension is 0, that is, there is no interfacial tension between the two phases in extreme cases, the viscous fingering phenomenon is more obvious, and there are obvious finger-like displacement in the area near the upper and lower plates. When the surface tension is increased to 7.2*mN/m*, there is no obvious finger-like appearance, but a distinct M-shape appears at the displacement leading edge, with protrusions near the upper and lower plate, which will develop into two finger-shaped. When the surface tension continues to increase to 14*mN/m*, it can be found that the whole is a relatively complete finger-shaped, with a smooth leading edge and no tendency to split. It can also be clearly seen from Fig 9 that the viscous finger subjects under different surface tensions travel to about 0.012m at 0.5s, and the biggest difference lies in the shape of their finger front ends. From the above results, it can be concluded that the interfacial tension has the effect of suppressing short waves, which is consistent with the results of Saffman & Taylor in Newtonian fluid [11]. It is used to analyze the effect of interfacial tension on interfacial stability, regular modulus perturbations are obtained exp(*σt*+*iky*) dispersion relations.


$$\sigma =\left[\frac{\mu_{1}-\mu_{2}}{\mu_{1}+\mu_{2}}U+\frac{(\rho_{1}-\rho_{2})gb^{2}}{12(\mu_{1}+\mu_{2})}\right]k-\frac{F_{st}b^{2}k^{3}}{12(\mu_{1}+\mu_{2})}$$ (7)

Among them, ***F***_*st*_ is the interfacial tension, which has the effect of suppressing short waves. The Capillary number has a great effect on viscous fingering, and it can be seen from Eq (8) that the Capillary number decreases as the interfacial tension increases, resulting in a decrease in the number of leaflets split at the fingertip [30].

$$C_{a}=\frac{v\mu }{F_{st}}$$ (8)

Where *μ* is the dynamic viscous with the unit of *P*_*a*_∙*s*,*ν* is the velocity at the fingering tip with the unit of *m*/*s* and ***F***_*st*_ is the surface tension with the unit of Nm.

**Fig 10:**
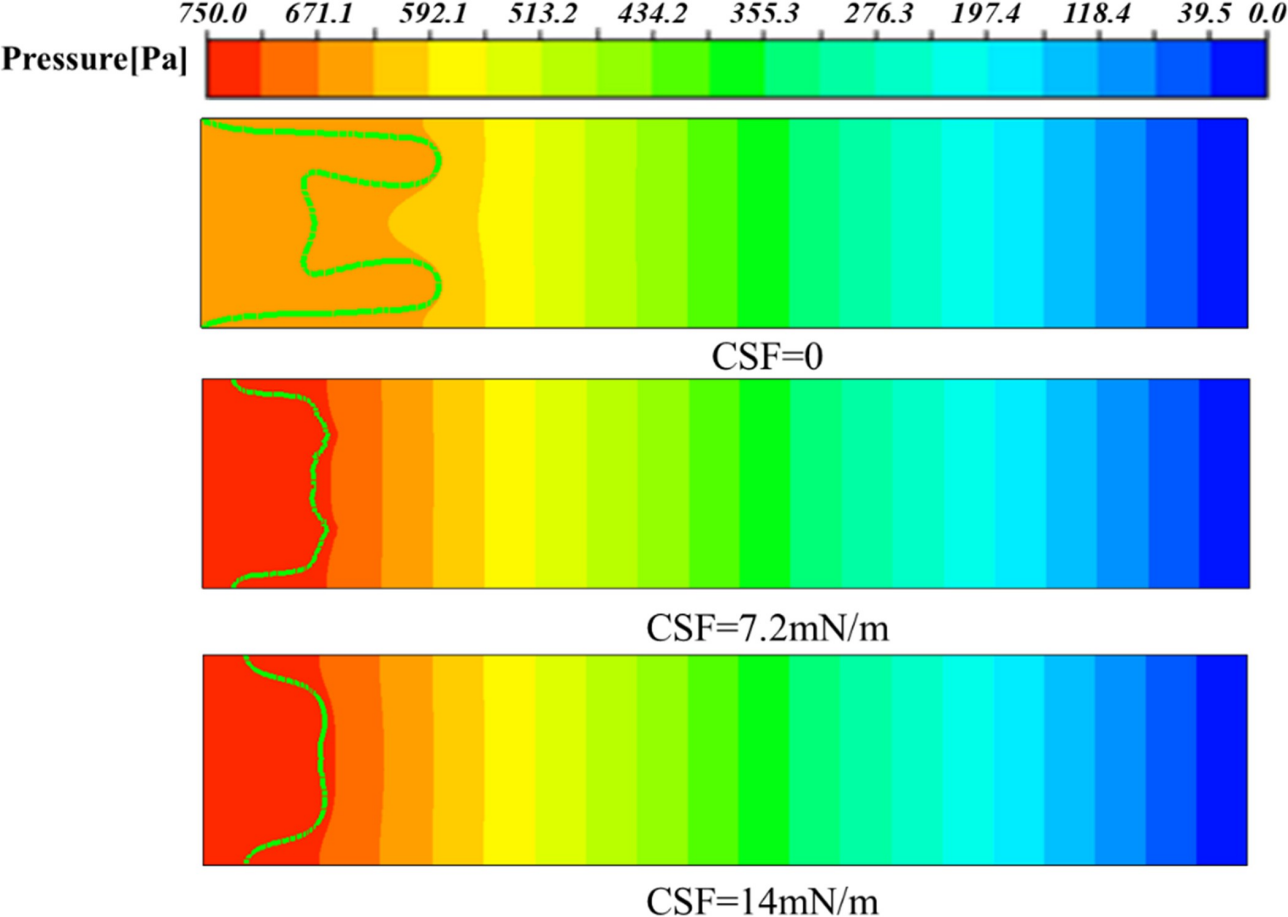
Contour of pressure distribution under different surface tensions at 0.5s.

**Fig 11:**
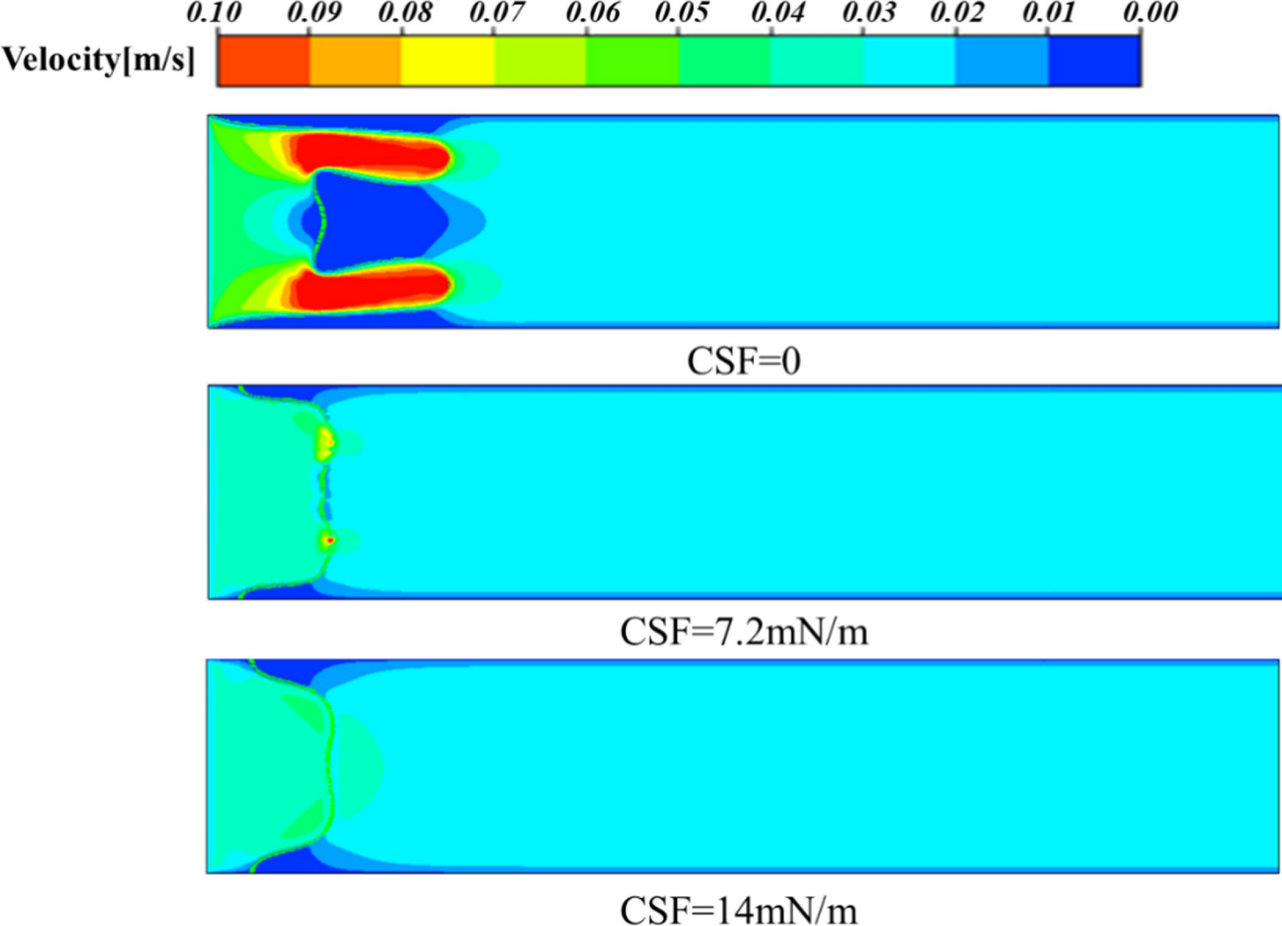
Velocity distribution at 0.5s under different surface tensions.

**Fig 12:**
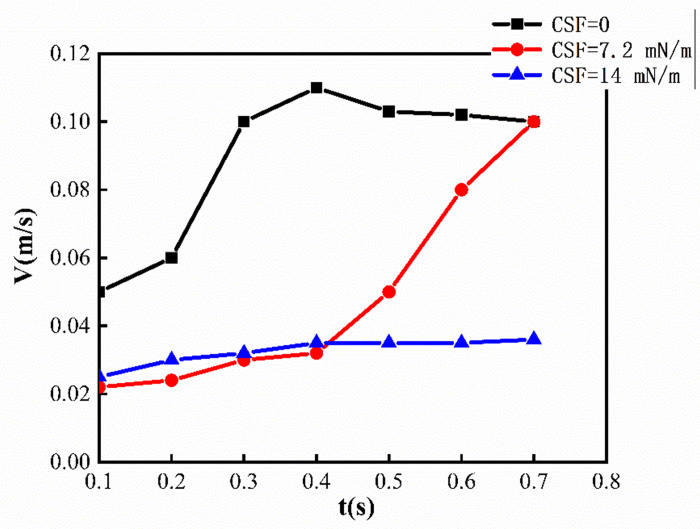
The velocity of the fingertip at different times.

This phenomenon can also be explained from the pressure distribution diagram, as shown in Fig 10, where the largest pressure gradient occurs at the tip of the viscous fingering, regardless of the change in interfacial tension, which will facilitate the finger movement further. In addition, it can be seen that when the interfacial tension is 0, the pressure value is the smallest, so its velocity is the greatest (as shown in Fig 11), and the finger travels the farthest. As can be seen from Fig 11, the maximum velocity occurs at the fingertip, whether the presence or absence of interfacial tension. When there is interfacial tension, the velocity is more evenly distributed in the fingers, while when the interfacial tension is 0, the velocity inside the two fingers is about twice that of the main part. In addition, when the interfacial tension is 0, the velocity of the fingertip is about 0.1m/s. When the interfacial tension is 7.2*mN/m*, the velocity of the fingertip is 0.07*m/s*, and when the interfacial tension is 14*mN/m*, the velocity of the fingertip is 0.04*m/s*. Therefore, as the interfacial tension increases, the speed of fingering into the tip decreases. We also extracted the velocity of the fingertip under different surface tensions at different times, as shown in Fig 12. Since after 0.7*s*, one of the finger advances at 0 interfacial tension starts to degenerate, so we extract the velocity value before 0.7*s*. When the interfacial tension is 0 and 7.2*mN/m*, the velocity has a large jump in 0.2*s* and 0.4*s*, because at this moment, the fingertip begins to split, the fingertip cross-section shrinks, and the velocity increases sharply. However, when the interfacial tension is 14*mN/m*, the velocity growth is relatively flat and basically maintained at 0.35*m/s* due to the absence of fingertip splitting, fusion, and other phenomena.

## 5. Effect of temperature on viscous fingering

**Fig 13:**
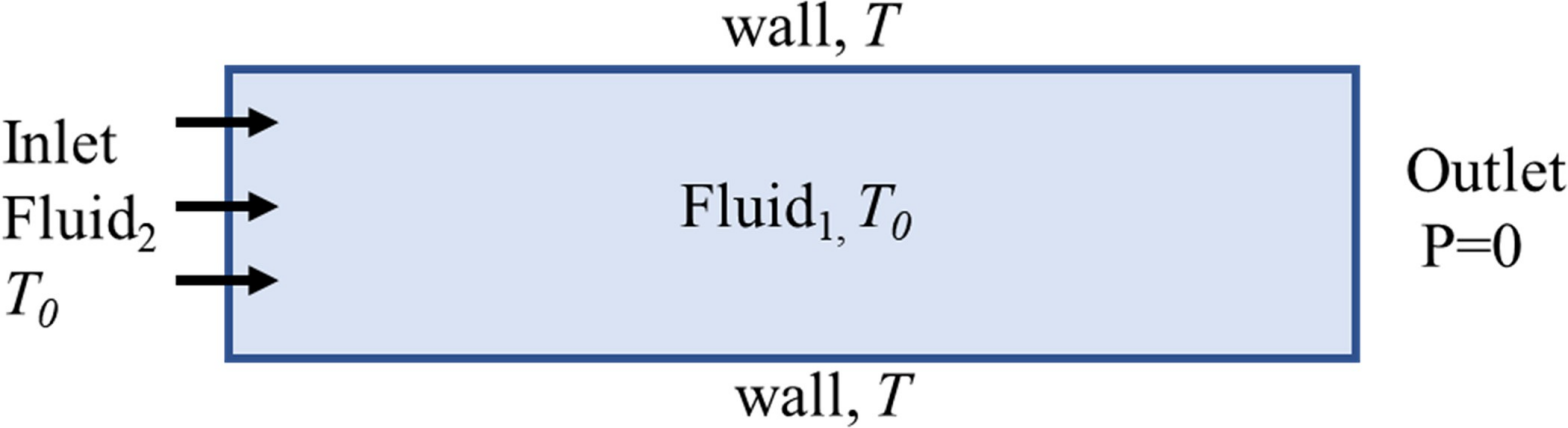
Calculation model considering temperature.

In industrial production, the presence of the displaced phase is influenced by environmental factors and seasonal changes, so its temperature is not constant. Therefore, it is necessary to consider displacement at different temperatures. In order to investigate the effect of temperature on viscous fingering, we conducted simulation studies at the temperatures of *T*_1_ = 300 *K*,*T*_2_ = 305 K, and *T*_3_ = 320 *K*, respectively. As shown in Fig 13, fluid 1 (non-Newtonian fluid) occupies the entire fluid domain at room temperature of 298.15 K, and then fluid 2 (air) flows in at room temperature of 298.15 K. We set the upper and lower walls to a non-slip wall with temperatures *T*_1_,*T*_2_, and *T*_3_ respectively, and the purpose of heating the fluid is achieved by maintaining a constant wall temperature.

In the calculation, the energy equation was opened, and re-set the viscosity of non-Newtonian fluids. The shear rate and temperature dependent model was selected, and the constitutive equation of viscosity is Eq (9). It can be found that the relationship between viscosity and temperature is inversely proportional, that is, the increase of temperature will lead to the decrease of viscosity.


$$\mu =\eta (\dot{\gamma })H(T)$$ (9)

Where *H(T)* is the temperature dependence, known as the Arrhenius law.

$$H(T)=exp\left[\alpha \left(\frac{1}{T-T_{0}}-\frac{1}{T_{\alpha }-T_{0}}\right)\right]$$ (10)

Where *α* is the ratio of the actication energy to the thermodynamic constant and *T*_*α*_ is a reference temperature for which *H(T)* = 1. T_0_ which is the temperature shift, is set to 0 by default and corresponds to the lowest temperature that is thermodynamically acceptable. Therefore *T*_*α*_ and *T*_0_ are absolute temperatures. Temperature dependence is only included when the energy equation is enabled Set the parameter *α* to 0 when you want temperature dependence to be ignored, even when the energy equation is solved.

**Fig 14:**
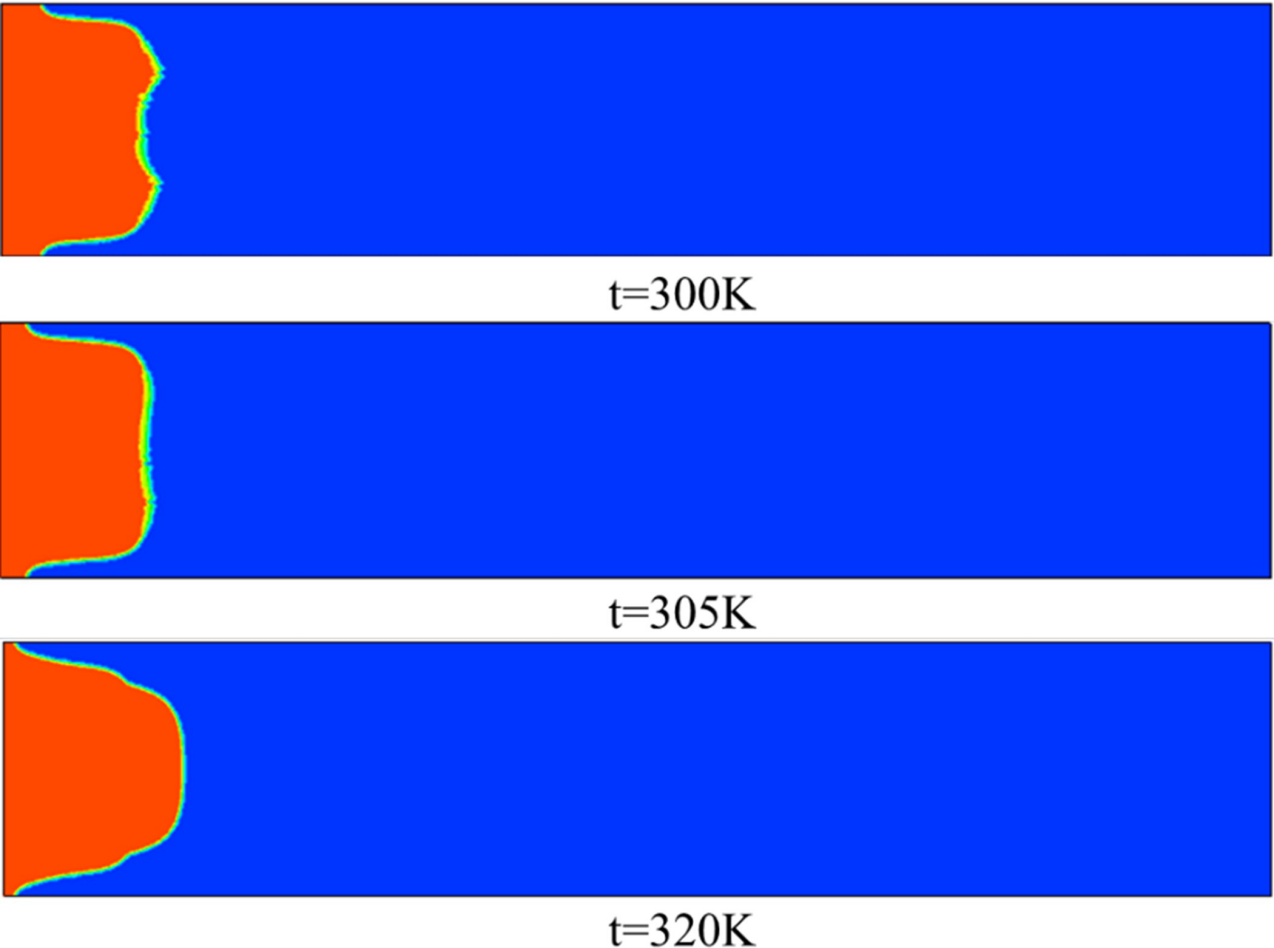
Viscous fingering under different temperatures at 0.5s.

**Fig 15:**
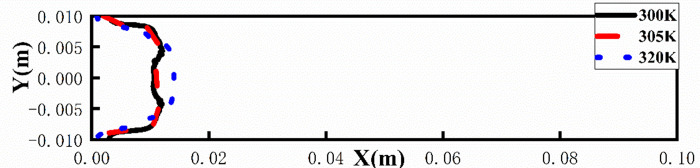
Position of viscous fingering at different temperatures at 0.5s.

**Fig 16:**
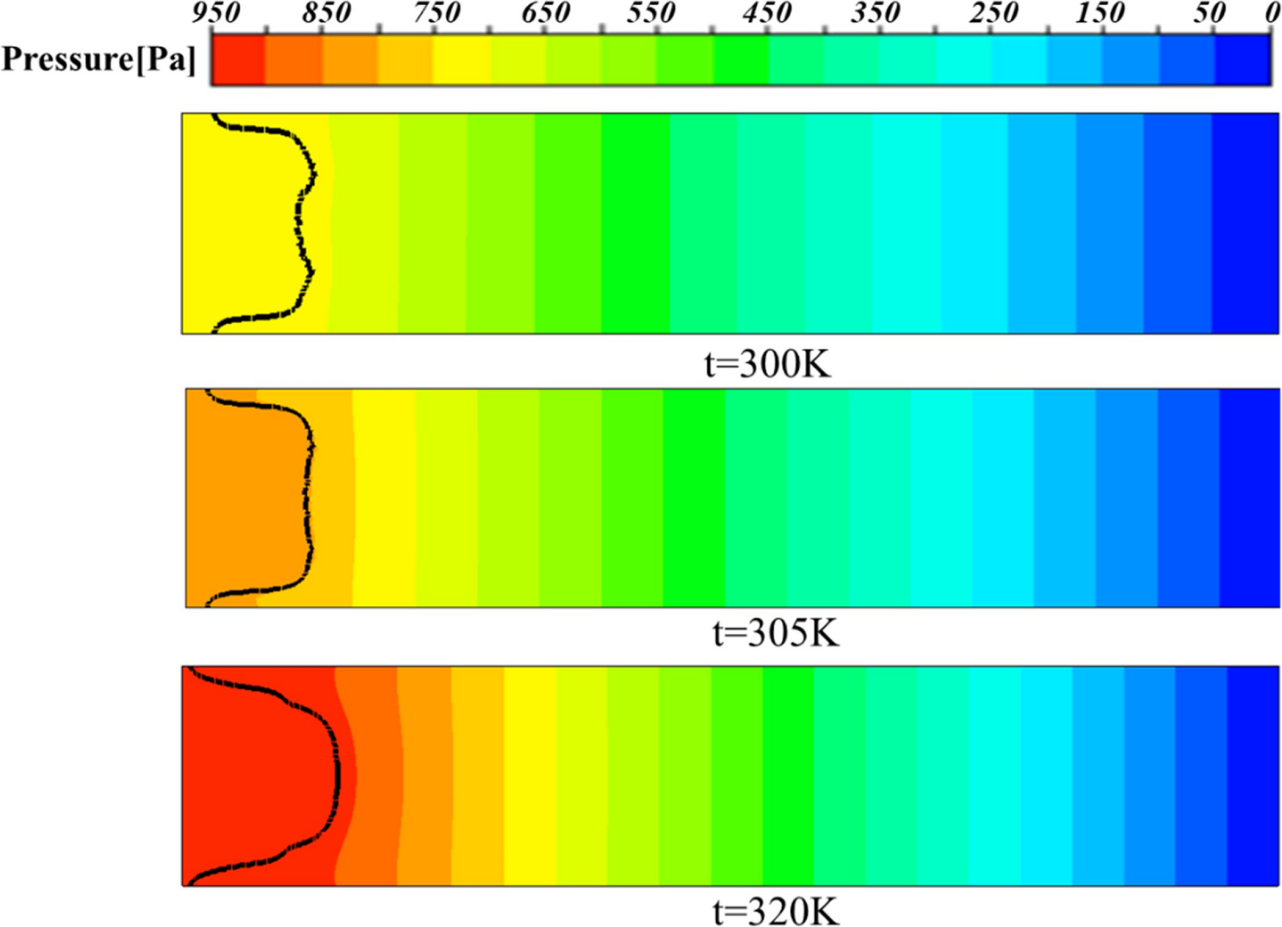
Pressure contour at different temperatures at 0.5s.

As shown in Fig 14, at a temperature of 300 K, a clear M-shaped interface appeared, with fingertips splitting at the tip and developing into two fingers. When the temperature increases to 305 K, it can be seen that the leading edge of the interface is relatively smooth, and there is no obvious M-type. When the temperature continues to increase to 320 K, it can be clearly seen from Fig 15 that the displacement distance is the farthest and the protrusion width is narrow. The results show that increasing the temperature can lead to a decrease in the viscosity of the displaced phase (a highly viscous fluid), making the flow more stable. In addition, from the pressure distribution of Fig 16, it can be seen that as the temperature increases, the pressure gradient inside the flow channel increases, so that at a temperature of 320K, the viscous finger moves further.

## 6. Conclusion

In this study, we used numerical simulation methods (ANSYS-Fluent) to investigate the viscous fingering of non- Newtonian fluids in the displaced phase of three-dimensional HSC. An analysis was conducted on different types of upper and lower surfaces, different interfacial tensions, and viscosity fingertips at different temperatures. The specific conclusions are as follows:

1. In the 3D model, when the upper and lower surfaces are set to walls, the finger passes split at the tip, and two fingers appear at the interface, with one fingertip developing relative to the other. This is the result of the combined effects of moving contact lines and Saffman-Taylor instability.
2. The existence of interfacial tension has the effect of suppressing short waves. In addition, when the interfacial tension exists, the distribution of velocity within the fingertip is relatively uniform, and as the interface tension increases, the velocity at the fingertip decreases. When the fingertip begins to split, the velocity increases sharply due to the shrinkage of the fingering interface. However, when the interfacial tension increases to 14*mN/m*, the velocity remains relatively stable due to the absence of fingertip splitting or other phenomena.
3. Raising the temperature will lead to a decrease in the viscosity of the displaced phase, making the flow more stable. And as the temperature increases, the pressure gradient inside the channel increases, and the viscous finger moves further.
